# Unraveling the Inferno: An Arson Case Series

**DOI:** 10.7759/cureus.60127

**Published:** 2024-05-12

**Authors:** Yarden Segal, Gurtej Gill, Garima Yadav, Gurraj Singh, Paulina Riess

**Affiliations:** 1 Psychiatry, BronxCare Health System, Bronx, USA; 2 Psychiatry, Texas Tech University Health Sciences Center, Permian Basin, Midland, USA; 3 Psychiatry, Sri Guru Ram Das Institute of Medical Sciences and Research, Amritsar, IND

**Keywords:** forensic psychiatry, psychiatric disorder, schizophrenia, arson, firesetting

## Abstract

Firesetting behaviors present multifaceted challenges that intersect the realms of mental health, law, and societal welfare. While distinct in nature, firesetting, arson, and pyromania collectively embody a spectrum of behaviors that cause harm with profound implications for mental health and legal system. Firesetting is a behavior, arson is a criminal offense, and pyromania is a classified psychiatric diagnosis. Nevertheless, the underlying motivations for these behaviors in the context of psychiatric symptomatoloty remain poorly understood. Arson alone exacts a staggering financial toll in the United States, emphasizing the urgent need to understand the reason behind these acts. Within legal contexts, mental health professionals frequently encounter individuals exhibiting firesetting behaviors while consulting on legal cases. The strong correlation between firesetting behavior and mental disorders shows a dire need for extensive and detailed collaboration between psychiatric experts, legal practitioners, and fire services. Here, we describe a series of firesetting cases and their ties to the mental health and legal systems underscoring the imperative for integrated, multifaceted approaches to address this pressing societal concern.

## Introduction

It is essential to distinguish between fire setting, arson, and pyromania due to their different implications. Firesetting is a behavior, arson is a crime, and pyromania is a psychiatric diagnosis [1]. Firesetting refers to the act of deliberately igniting fires, regardless of the individual’s intent or motivation [2]. This can encompass both accidental or purposeful acts [2]. Arson, a subtype of firesetting, is a criminal act in which a person or group of persons willingly and maliciously sets fire or aids in firesetting to cause harm to property, people, and infrastructure, and by definition, excludes accidental firesetting [1,2]. Arson is a serious offense with legal consequences and is prosecuted under criminal law [2]. Pyromania is a psychiatric disorder characterized by an uncontrollable urge to set fires [2]. Furthermore, it is a repeated, deliberate, purposeful firesetting associated with tension or affective arousal before the act, followed by intense pleasure or relief when setting the fire or witnessing its aftermath.

The onset of this behavior is usually in childhood and may continue through adolescence and adulthood with increasing frequency and intensity of firesetting over time [1,2]. In children and adolescents, firesetting behaviors have been associated with mood disorders, anxiety disorders, conduct disorders, oppositional defiant disorders, and attention deficit disorders [3]. Most arsonists are male, but the proportion of females causing arson is on the rise [4]. The differentiation between these behaviors is essential for the legal system and mental health system, as well as for public safety and fire prevention purposes. In the criminal legal system, distinguishing between the terms is crucial for defining criminal offenses, determining culpability, and determining appropriate legal consequences [1,2]. In the realm of mental health, the distinctions are essential for accurate diagnosis, intervention, and treatment strategies [1,2].

Deliberate firesetting or arson is a huge problem worldwide; it causes substantial damage and significantly impacts public health and safety. In the United States, 64,000 cases were reported in 2007, corresponding to 25 offenses per 100,000 individuals [5]. In Australia, arson is estimated to cost around $1 billion in damages per year, and there is an increase in wildfires that have been started deliberately [5]. In the United Kingdom, arson costs about £2.8 billion annually; an estimated 3600 fires were started deliberately every week in 2007, and around 40,000 offenses were recorded by police [5].

The motive behind arson is significant, and most studies have considered revenge and hate as essential motives [6,7]. According to Lewis and Yarnell, arson can be classified into five groups: committed by children, accidental, as an expression of hatred and revenge, erotic, and in a state of hallucination or delusion [6]. Children were often found to engage in firesetting behaviors due to curiosity, peer pressure, or experimentation. They often lacked an understanding of the consequences of their actions, distinguishing this group from the other categories of arson [6]. Accidental firesetting involved situations where fires were ignited unintentionally, often due to negligence mishaps, or human error. Arson committed as an expression of hatred or revenge was typically aimed at specific individuals, properties, or organizations perceived as enemies or adversaries [6]. Erotic firesetting cases involved individuals who derived sexual arousal or gratification from the act [6]. Firesetting behaviors that occur in the context of delusions and hallucinations typically involve individuals with a distorted perception of reality that influences their behaviors leading to firesetting acts [6]. Furthermore, Fleszar-Szumigajowa found that 53 percent of his sample had revenge, and 33 percent had hallucinations and delusions as motives for firesetting [7].

Studies have revealed an association between arson and mental disorders such as substance use disorder, personality disorders, neurotic spectrum disorder, intellectual disability, mood disorders, and schizophrenia [8]. According to Yesavage et al., 10 percent of all convicted arsonists had schizophrenia [8]. He reported that 54 percent of arsonists had a diagnosable mental illness, and mentally ill arsonists set more fires than the non-mentally ill group [8]. Ritchie and Huff concluded that 90 percent of arsonists had histories of mental health issues [4].

The likelihood of an arson offender having schizophrenia is 20 times greater than that in the general population [5]. Schizophrenia and borderline personality disorder are the most common diagnoses in mentally disordered individuals. Geller and Barnett et al. found that individuals with schizophrenia usually set fires under the influence of their psychiatric symptoms [9,10].

The strong correlation between firesetting behavior and mental disorders demonstrates a dire need for extensive, detailed collaboration between psychiatry, legal expertise, and fire services.

## Case presentation

Case one

The patient was a male in his 50s diagnosed with schizophrenia with an extensive history of over 50 inpatient hospitalizations in the context of treatment non-compliance. His history included five suicide attempts and numerous years of Assertive Community Treatment (ACT) team follow-up in conjunction with court-mandated community treatment. 

The patient was admitted to a secure facility for restoration to competency on charges of arson in the second, fourth, and fifth degrees as well as reckless endangerment in the second degree. These charges were based upon allegations that he was observed standing on the threshold of a neighbor's apartment, where he allegedly set fire to a pile of clothing, ultimately attempting to set fire to a 14-story apartment building. He was psychotic and evidenced profound delusions of paranoia at the time, subsequently believing that he performed the alleged crime in self-defense. 

The patient was found incompetent to stand trial due to active symptoms of schizophrenia. Two forensic evaluators concluded that his symptomatology (grandiose and paranoid delusions as well as auditory, visual, and tactile hallucinations) interfered with his ability to possess a factual and rational understanding of the legal situation and basic courtroom procedure. Ultimately, these factors interfered with his ability to assist counsel and participate in his defense. At the time of his assessment, he specifically presented with grandiose delusions, including owning the building in which he was accused of setting a fire, having many high-powered jobs, and having personal relationships with many celebrities. He was adamant that his charges would be dismissed because of his many professions, including being a lawyer, firefighter, and entertainer. Furthermore, he was internally preoccupied, responding to internal stimuli in an acutely psychotic manner.

The patient had a long history of being prescribed multiple psychotropic medications including multiple first-generation and second-generation anti-psychotics as well as mood stabilizers. He was ultimately treated with medication for treatment-resistant schizophrenia, namely clozapine. 

While the patient had long-standing delusions and auditory hallucinations, he had never previously attempted to harm others in the community. He had instances of self-harming behavior, including stabbing himself in the abdomen and overdosing on psychotropic medication leading to ICU admission and subsequent hospitalization in an inpatient psychiatric unit. He did present with multiple episodes of psychotic agitation during this hospitalization, necessitating the administration of emergency intramuscular medication. He had no previous history of fire-setting behavior. After the detainment, he restarted his past regimen of clozapine, haloperidol decanoate, and valproic acid. Although he no longer experienced hallucinations, he remained profoundly paranoid and was unable to be restored to competency for over one year. His charges were ultimately dropped by the court, and he was transferred to a non-secure long-term facility for further treatment. 

Case two

The patient was a male in his 30s diagnosed with schizophrenia and conduct disorder with an extensive psychiatric history of repeated violent offenses and hospitalizations due to delusions and auditory hallucinations resulting from treatment non-compliance.

According to police records and eyewitnesses, the patient set two men on fire inside a liquor shop by throwing a lit alcoholic bottle toward them while trying to put the shop ablaze. He also stabbed a bystander in the back of the head and slashed her face with a knife. The patient was subsequently charged with arson in the first and second degrees, murder in the second degree, attempted murder in the first and second degrees, assault in the first and second degrees, and resisting arrest. 

At the time of the offense, the patient was paranoid and delusional with auditory command hallucinations. During his hospitalization, he exhibited disorganized thinking (i.e., confusing, rambling, digressive, and overly detailed), poor insight, and a lack of remorse and responsibility for the criminal act. 

He accepted the plea of Not Responsible Due to Mental Disease and was admitted to a forensic psychiatric center for an inpatient examination of his dangerousness. During the evaluation, he still exhibited active symptoms of psychosis and he could not appreciate the wrongfulness of his conduct at the time of the offense, and his delusions that one can be murdered, yet still function and appear to be alive, undermined his capacity to appreciate the nature and consequences of his behavior. Ultimately, examiners opined that he suffers from a dangerous mental disorder (DMD), a legal term used to describe a mental illness that presents significant harm to oneself or others. The court concluded that he required ongoing care and treatment in a secure inpatient psychiatric facility.

The patient had a long history of being prescribed psychotropic medications. He was eventually maintained on clozapine 350 milligrams daily and fluoxetine 40 milligrams daily with partial response. He continues to have paranoid ideations and poor insight into his mental illness despite medication compliance.

Case three

The patient was a male in his 40s with a well-established diagnosis of schizophrenia, multiple suicide attempts, and chronic auditory hallucinations and delusions. He had an extensive history of repeated hospitalizations due to either treatment non-compliance or incomplete response to medication. Due to his chronic non-compliance, he was placed on an Assisted Outpatient Treatment (AOT) order and with an Intensive Case Manager (ICM) before the instant offense.

On the day of the instant offense, he dispersed lighter fluid throughout his assigned accommodation at the adult care home and lit the room on fire while his roommate was inside. Extensive damage ensued throughout the room, including the beds, walls, ceiling, and other personal belongings. The roommate subsequently was treated for smoke inhalation; the patient was charged with arson in the second degree and additional related charges. He later acknowledged having intentionally lit the fire to eliminate command auditory hallucinations that had been bothering him for three years before the instant offense.

The patient accepted a plea of Not Guilty because of Mental Disease or Defect to the charges of arson in the second degree and related offenses on the condition of medication compliance. Criminal Procedure Law (CPL) 330.20 examiners opined that he suffered from a DMD and required treatment in a maximum-security inpatient psychiatric facility due to his history of impulsivity, violence, and supervision failure. He was ultimately maintained on clozapine 500 milligrams HS (taken at bedtime) and long-acting injectable paliperidone 234 milligrams intramuscularly monthly. However, he remains with residual symptoms of chronic auditory hallucinations and paranoid ideations.

Case four

The patient was a female in her 20s diagnosed with a schizoaffective disorder characterized by significant mood symptoms, delusions, and command auditory hallucinations. She also had a medical history of multiple sclerosis (MS). She had a history of repeated hospitalizations for multiple suicide attempts, violent outbursts, and repeated fire-setting behaviors.

The patient was hospitalized for setting her house on fire and running naked through the streets. She endorsed experiencing commanding auditory hallucinations and paranoia during the initial act of arson, including voices telling her to kill herself. The patient improved with treatment and was discharged to outpatient care but without full resolution of her symptoms. Over a few months, in context to medication non-compliance, the patient again began perpetrating arson-like behavior, starting multiple fires in her hotel room, the trash bins in the hotel stairway, and her rental car windshield, causing significant property damage. She again reported this was in the context of commanding auditory hallucinations and paranoid ideations. She was subsequently charged with attempted arson in the second degree, criminal mischief in the second degree, and arson in the third degree.

The patient was evaluated and deemed fit to stand trial after noting an improvement with anti-psychotic medication during hospitalization with a resolution of acute hallucinations, paranoia, and delusions. She presented without impairment of her ability to learn and understand the charges against her. It was indicated that the patient's medical diagnosis of MS or the treatment use of steroids possibly contributed to her psychiatric presentation. She accepted a plea of Not Responsible Due to Mental Disease and was admitted to a forensic psychiatric center for an inpatient examination of her dangerousness. Examiners opined that she suffered from a DMD and required ongoing care and treatment in a secure inpatient psychiatric facility due to numerous psychiatric hospitalizations, non-compliance, rapid decompensation, setting fires in the context of psychosis, and poor insight into her psychiatric illness.

Ultimately, she was maintained on risperidone 4 milligrams daily for psychosis, lithium 900 milligrams daily for mood stabilization, and teriflunomide for her MS. She remains with residual symptoms of her illness.

Case five

The patient was a male in his 50s diagnosed with schizoaffective disorder, antisocial personality, and alcohol use disorder while experiencing symptoms of paranoia and delusional thoughts. He had a long-standing history of numerous inpatient hospitalizations, repeated arrests for violent behavior, and months of being followed by an ACT team. 

The patient was observed entering his rental residence and setting his roommate's bed on fire, with whom he often had disagreements. He was subsequently charged with arson in the second degree and deemed psychotic along with polysubstance abusive and classified as seriously and persistently mentally ill.

During the initial psychiatric assessment, the patient lacked insight into the importance of strict medication adherence and abstinence from substance use, demonstrating a limited understanding of how alcohol use could exacerbate his clinical symptoms regardless of medication compliance. The patient accepted a plea of Not Responsible Due to a Mental Disease or Defect. He was subsequently determined to have a DMD, requiring care and treatment in a secure psychiatric facility.

The patient was maintained on olanzapine 35 milligrams daily and continued to adhere to the medication prescribed with good response. He has had some resolution of his symptoms, including problems with impulse control; however, he remains with residual symptoms of psychosis. 

## Discussion

Arson and firesetting behavior are significant worldwide issues, causing damage and destruction. Just in the United States, there are over 62,000 arsons committed annually, with nearly $1 billion in losses per year [11]. A study by Vaughn et al. showed that the prevalence of firesetting in the United States was 1.0 percent [12]. A single fire can cause extensive physical, social, and economic harm to society. According to the Uniform Crime Reports of the Federal Bureau of Investigation (FBI), arson cases have continued to rise from 2015 to 2016 by 2.3 percent [11].

Several studies have demonstrated gender differences among those who set fires. Most arsonists are male, but the proportion of females causing arson is on the rise [4]. Compared to male firesetters, females were found to have higher levels of depression and psychosis [13]. Female arsonists mostly had a co-morbid borderline and antisocial personality disorder diagnosis [13]. Male arsonists were more likely to have co-morbid diagnoses such as substance use disorder, conduct disorder, oppositional defiant disorder, antisocial personality, and schizoid personality disorder [14]. Furthermore, the literature estimates that 13.6 percent to 17 percent of teenagers engage in firesetting behavior [14]. It is commonly seen in children with histories of sexual abuse, family dysfunction, school difficulties, substance use disorders, and personality traits such as hostility and impulsivity [14].

Anger, hostility, and impulsivity play crucial roles in firesetting behavior among juveniles [15]. In children, firesetting is associated with depression, conduct disorder, oppositional defiant disorder, attention deficit hyperactivity disorder (ADHD) symptoms, and cruelty to animals [15]. These children generally have poor judgment, poor social interaction, feelings of loneliness, hopelessness, and peer rejection [3]. Therefore, it is vital to screen such patients at an exceedingly early age going back to their childhood and learn about their interest in fires and firesetting behavior. Currently, no available questionnaire can help screen individuals for fire-setting behavior. Developing such a questionnaire is vital, as it will help identify individuals with a propensity for fire setting and would help mitigate their risk of engaging in such behaviors. and that could help prevent arson.

The literature shows that the motive behind arson is critical and differs between mentally ill and non-mentally ill offenders. Revenge is a motive common to both mentally ill and non-mentally ill offenders. However, excitement, attention seeking, communicative arson, suicide attempts, and vandalism are primarily seen in the mentally ill. Rix found that the motives of mentally ill firesetters are more complex and commonly have more than one motive [16]. Furthermore, he noted that vandalism appears to be strongly associated with non-mentally ill firesetters [16].

Communicative arson is setting fire to communicate a desire, wish, or need. Mentally ill firesetters are commonly associated with communicative arson. They typically attempt to express their desire to change their institution, the location of services provided, or a wish to return to the hospital. Geller found that communicative arson is a channel of expression for mentally ill firesetters [9].

According to Ritchie and Huff, if the motive for firesetting in mentally ill individuals is revenge, they are likely to choose an individual or an organization with which they are angry [4]. They might set fires to their dwellings, the houses of spouses, relatives, and acquaintances [4].

The literature points to a strong association between firesetting and antisocial behavior. Examples include shoplifting, cutting class, robbing, mugging, and destruction of property [12]. There is also an increased frequency of antisocial behaviors in those with a lifetime history of firesetting [12]. Individuals with mental illness or substance use often carry out fire setting [5,8]. A strong association was seen between lifetime alcohol use, marijuana use, conduct disorder, obsessive-compulsive personality disorders, antisocial personality disorder, and having a family history of antisocial behavior [12].

Authors of a Swedish case-control study placed arson in the same category as homicide, as both crimes are strongly associated with psychotic disorders. The study found that the arsonists were more likely to have a diagnosis of schizophrenia [5]. A recent study showed that 90 percent of arsonists had documented histories of mental illness. The authors found that 36 percent had schizophrenia, and 64 percent engaged in drug and alcohol use during arson [4].

According to Grant and Kim [17], substance use disorder, particularly alcohol use, was found to be prevalent among mentally disordered firesetters. Dickens et al. found that 62 percent of mentally disordered male arsonists were under the influence of alcohol when setting the fire [18]. As seen with the study of Grant and Kim [17], our fifth case patient had alcohol use disorder and polysubstance use with a diagnosis of a schizoaffective disorder, which contributed further to worsening his symptoms and causing the firesetting despite medication compliance. Therefore, screening individuals with arson offenses is essential as they often have co-morbid alcoholism.

One factor that plays a vital role in firesetting, especially among psychiatric patients, is medication non-compliance. As seen in all our cases above, the patients were not taking medication, leading to their symptoms’ aggravation and increased firesetting behaviors. This impulsivity and tendency to set fire can be decreased if the patients comply with their medication regimen and there is no co-morbid substance abuse. It is crucial for mental healthcare workers and family members of such patients to always keep a check on their medication. Richard et al. found that alcohol and drugs have a disinhibiting effect on an individual and further act as facilitators for actions like arson, in which they may not indulge in sobriety, which is further worsened with not being on psychiatric medication [19]. There is a lack of literature on the non-compliance of medication in people with schizophrenia and their impulsivity to set fire. More extensive research and studies need to be conducted to know the exact reason for arson and firesetting in psychiatric patients, especially people with schizophrenia.

Many fires can be controlled by collaboration between mental health workers and fire service workers. An example is TAPP-C (The Arson Prevention Program for Children) [20]. This program provides children with education and mental healthcare [20]. Firesetting behavior that leads to arson is often chronic. Collaboration between community organizations like mental health teams, insurance companies, schools, children's protective agencies, the legal system, and the court is essential for identifying, treating, and preventing firesetting behavior [3].

## Conclusions

The compilation of these cases sheds light on the intricate interplay between severe mental illness and criminal behavior, navigating challenges in legal proceedings and determining culpability. Individuals diagnosed with schizophrenia display varied manifestations, resulting in a spectrum of legal outcomes and implications for treatment. Common threads include non-compliance with treatment, recurrent hospitalizations, and differing levels of violence. These cases highlight several commonalities among the patients: all had a diagnosis on the psychotic spectrum, a history of multiple hospitalizations, chronic treatment non-compliance, and experienced auditory hallucinations and paranoia at the time of their crimes. Most of the patients in the cases had failed higher levels of outpatient treatments in the community such as court-mandated treatment, assertive community treatment, and intensive treatment teams.

Furthermore, due to their mental illnesses, all were found to be not guilty by reason of insanity, requiring treatment in secure forensic psychiatric facilities. Additionally, cases one, three, and four had a history of multiple suicide attempts, while cases two and four had a history of firesetting behaviors, and case five had a significant history of alcohol use. These cases underscore the necessity for nuanced, multidisciplinary approaches that address psychiatric symptoms, medication adherence, and comorbidities. Legal considerations should encompass mental states and competency and are crucial in determining culpability and appropriate legal outcomes. These cases further emphasize the importance of collaborative efforts for comprehensive care, early intervention, and understanding psychiatric influences on arson. This requires collaborative actions across multiple sectors, including mental health services, law enforcement, criminal legal systems, and community organizations to effectively prevent, manage, and mitigate the destructive consequences of firesetting behaviors.
